# Case of maxillary actinomycotic osteomyelitis, a rare post COVID complication-case report

**DOI:** 10.1016/j.amsu.2022.104242

**Published:** 2022-07-31

**Authors:** Wajiha Arshad, Mahjabeen Mahmood Kamal, Zunaira Rafique, Manahil Rahat, Hassan Mumtaz

**Affiliations:** a Consultant Radiologist, KRL Hospital Islamabad, Pakistan; b Resident Radiologist, KRL Hospital Islamabad, Pakistan; c Resident Oral Pathology, Armed Forces Institute of Pathology, Pakistan; d Clinical Research Associate, Maroof International Hospital Islamabad, Public Health Scholar, Health Services Academy Islamabad, Pakistan

**Keywords:** Osteomyelitis, Actinomycosis, CT scan, MRI

## Abstract

**Introduction:**

and Importance: Maxillary actinomycosis is a persistent, very rare disease produced by Actinomyces species which may include only soft tissue or bone or the two together. Actinomycotic osteomyelitis of maxilla is very infrequent when compared to mandible.

**Case presentation:**

Here we are conferring a case of an elderly male patient who had history of COVID-19 infection 4 months ago, with constant complaint of non-remitting vague pain in the region of maxilla with tooth loosening and extractions. He was given a provisional diagnosis of chronic osteomyelitis of maxilla which was later on proved by histopathology as actinomycotic osteomyelitis.

**Clinical discussion:**

A saprophytic fungus causes mucor mycosis, and it is quite unusual. Strawberry gingivitis is one of the signs and symptoms. Mucormycosis and post-covid oral maxillofacial problems can be improved with early diagnosis. Oral Mucormycosis should be suspected in individuals with weakened immune systems, uncontrolled diabetes or post-covid instances. Surgery and adequate antibiotic treatment are necessary to treat actinomycosis. Infection may return after a period of inactivity, so long-term follow-up is necessary.

**Conclusion:**

We conclude a positive causal association between COVID-19 and actinomycosis. Maxillary osteomyelitis, a very rare infection, and in our case, the causative organism was Actinomyces Patients who have been infected should be tested for Actinomycin, which may masquerade as a head and neck illness.

## Highlights

• Osteomyelitis caused by fungus is a potentially fatal opportunistic illness that is extremely rare. The orofacial region is usually affected, particularly the nose and paranasal sinuses. It's a dangerous infection that must be treated right away if it isn't to become fatal.
• Thrombosis and necrosis of adjacent hard and soft tissues occur as a result of vascular tissue invasion upon inhalation of the pathogen. Infection begins in the nose and paranasal sinuses and progresses to the lungs.
• We Present an old male patient with history of COVID-19 infection with chronic non-remitting nonspecific discomfort in the region of maxilla with teeth loosening and extractions.
• Histopathology later revealed that he had actinomycotic osteomyelitis, which was initially diagnosed as chronic maxillary osteomyelitis.

## 1 Introduction

**Fig. 1:**
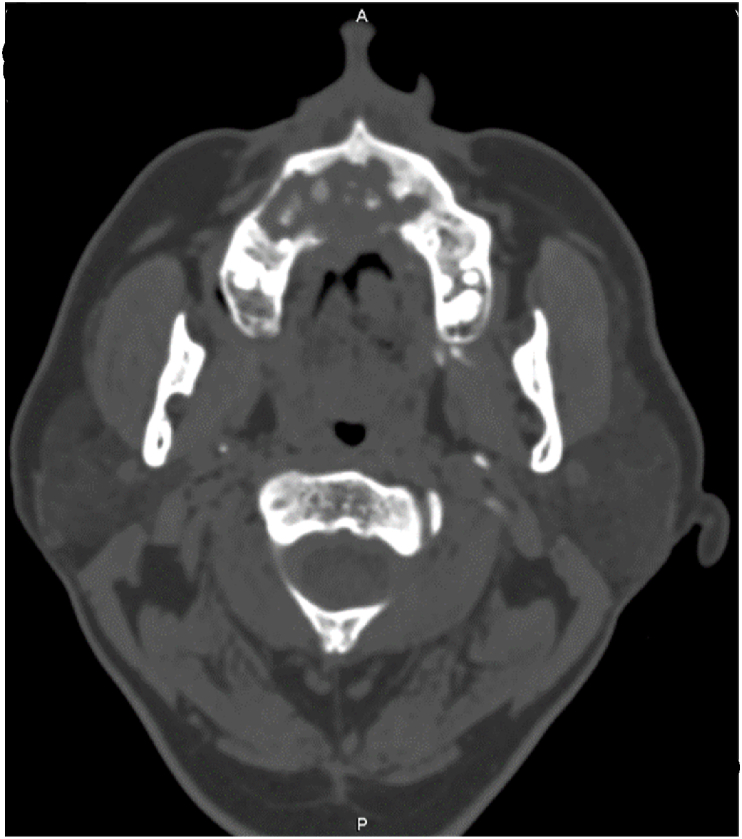
“Actinomycosis involving the maxilla in a 56-year-old man. Axial contrast-enhanced CT image (bone window) shows irregular erosive bone destruction with irregular fragments of dense bone in the center of lysis, findings indicate chronic osteomyelitis”.

**Fig. 2:**
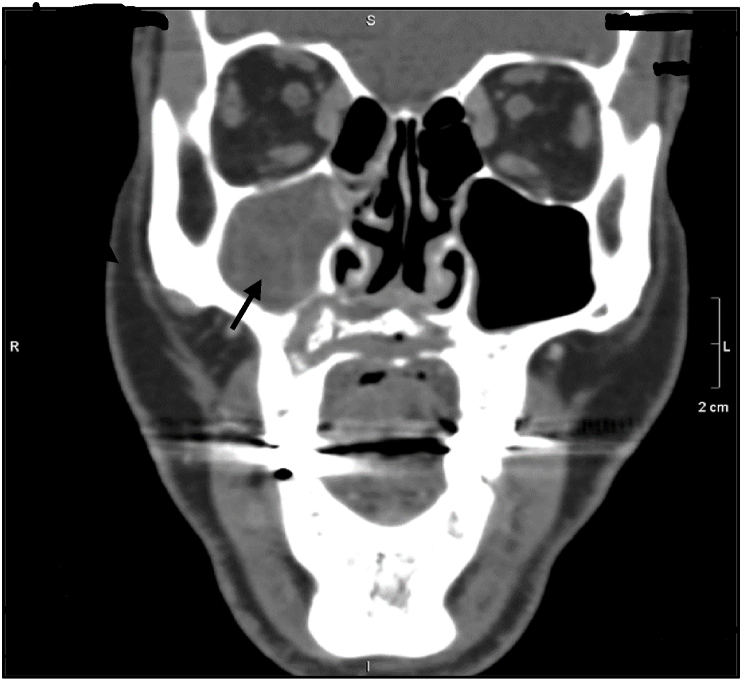
Coronal CT image showing lytic lesion in maxilla with central sequestrum.

**Fig. 3:**
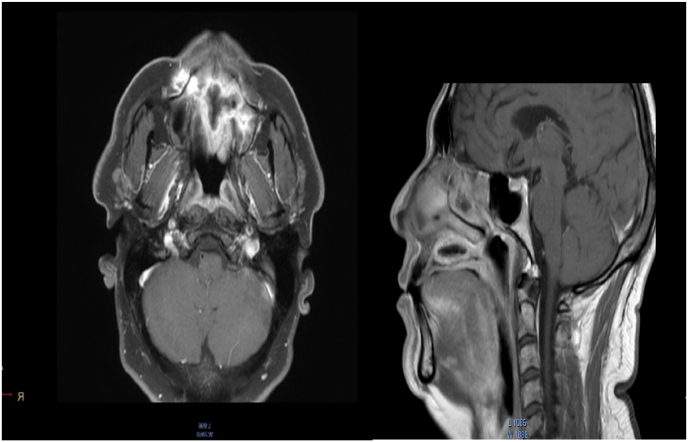
Showing MRI with contrast showing marginal enhancement of the maxilla with central necrosis. No significant soft tissue component and cervical lymphadenopathy is noted.

**Fig. 4:**
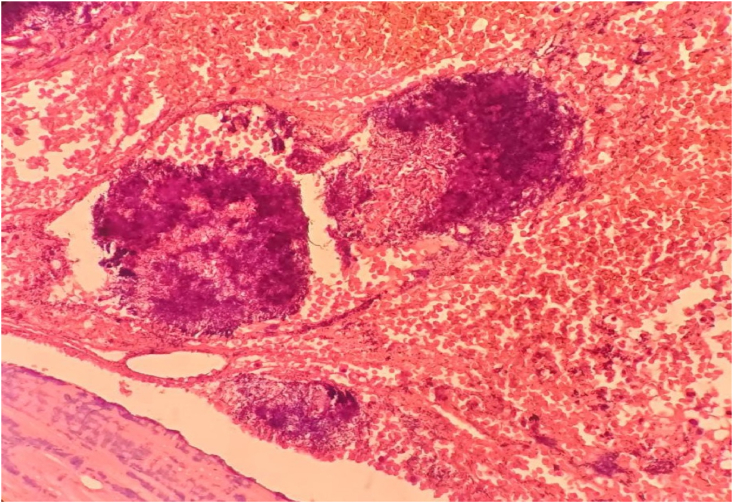
Gram stain showing filamentous bacteria.

Resident oral microorganisms, Actinomycete, are the primary cause of Actinomycosis, an extremely rare saprophytic infection. Actinomyces are fungi-like filamentous bacteria. They are Gram-positive, non-acid fast, anaerobic or microaerophilic bacteria that grow slowly. A. israelii, A. viscosus, A. odontolyticus, A. naeslundii, or A. meyeri are the most common species isolated from actinomycotic lesions [1] (see Fig. 1, Fig. 2, Fig. 3, Fig. 4).

With a ratio of 3:1.1, it exhibits a masculine predominance. Only seldom does bone get involved in the disease's pathology. Because the maxilla has a greater blood supply than the mandible, it is less prone to be infected with actinomycotic osteomyelitis. Actinomycosis usually causes abscess formation, dense fibrosis, and draining sinuses and sinus tracts that may discharge ‘Sulphur granules [2]. A rare kind of saprophytic bacterial infection, actinomycosis is often caused by the anaerobic Gram-positive bacterium Actinomyces Israel. The risk of Actinomycotic infection is increased by variables such as the disruption of the oral mucosa and/or systemic causes such as diabetic mellitis or other immunocompromised conditions [3].

Acute maxillary osteomyelitis has decreased in frequency since antibiotics were introduced. Odontogenic infections and sinusitis are the two most common causes. There is an increased risk of osteomyelitis in patients with diabetes, HIV, and malnutrition. It's critical to catch this early on because it can have catastrophic implications if left untreated [4].

The mortality rate of Mucor mycosis varies from 10% to 100% depending on the region of infection and the underlying predisposing factors, according to published literature. If you're diagnosed with maxillary sinus Mucor mycosis, you're going to die. When it comes to children, aspergillosis carries a mortality rate of 30%, with a mortality rate as high as 85%. Such patients require prompt diagnosis and treatment from the outset. The importance of surgical or prosthetic rehabilitation (RECONSTRUCTION) and surgical or antifungal therapy, surgical debridement, supportive therapy, and control of the underlying risk factors in treatment (RECONSTRUCTION) cannot be overstated [5].

About 0.5–9% of all occurrences of head and neck cancer are caused by the parasite Maxillary Actinomycosis. Few instances of primary maxillary actinomycosis have been documented in the literature [6]. We present a case of actinomycosis resulting in significant maxillary destruction, where the patient had diabetic mellites and a recent Covid infection that was treated with large doses of I/V steroids.

### 1.1 Case Presentation

Reporting on a 56-year-old male patient at KRL Hospital Islamabad with a history of diabetes mellitus and hypertension. He developed COVID-19 infection four months ago and was treated with heavy doses of I/V steroids. In his post treatment phase, he started having vague facial pains, discomfort and tooth loosening for which he endured multiple tooth extractions involving the superior and inferior alveolar arches.

On examination no cervical lymphadenopathy was noted. The patient was then inspected with OPG and a CT scan of his face. The Consultant Radiologist reported having an erosive pattern of maxillary lysis with irregular marginal sclerosis and dense bone in the center of the lytic area giving a sequestrum like appearance. No associated soft tissue mass or aggressive bone destruction noted. The CT report indicated the appearances indicative of chronic osteomyelitis.

Keeping in view the absence of lymphadenopathy and the other CT features of the disease kept the possibility of a malignant process less likely. MRI with contrast was also done showing intense marginal enhancement of the eroded maxilla with a central signal void sequestrum further endorsed the CT findings.

Bone and soft tissue samples were sent for histopathologic evaluation after the patient underwent surgical debridement, and the results showed necrotic bone with mixed inflammatory infiltrate and granulation tissue, as well as actinomycete colonies permeating the bone tissue and containing colonies of actinomycotic organism. Club-shaped filaments are formed by the rosette-shaped colonies with basophilic strands on the periphery and eosinophilic club endings. An actinomycotic osteomyelitis diagnosis was established.

In the subsequent follow-up appointments, the patient is responding well to antimicrobial drugs and is exhibiting signs of improvement.

Our Case Report is in compliant with SCARE 2020 Guidelines [7]. A complete SCARE 2020 checklist has been provided as a supplementary file. Our study has been registered on Research Registry with the following UIN: researchregistry8109 [8].

## 2 Discussion

The presence of fungus. A saprophytic fungus causes mucor mycosis, and it is quite unusual. (phycomycetes). There are a number of early clinical indications include nasal crusting and stuffiness alone or with complaints of perinasal paresthesia and periorbital cellulitis, as well as arthralgia and weight loss. As the infection progresses, there may be eschars and necrosis in the nano-facial area as well. Strawberry gingivitis is one of the signs and symptoms. Even in the absence of death, severe infection may cause thrombosis of the internal jugular vein or carotid artery [9].

Osteomyelitis of the facial bones is rare because of the maxilla's high vascularity. Although the use of broad-spectrum antibiotics has decreased the number of cases, the illness persists in countries with poor health systems and those with low incomes. Osteomyelitis insipida due to a fungus is very rare [10].

Thrombosis has rendered medical therapy useless because of the lack of medication concentration and access to the infection site, making it impossible to cure the disease. Controlling diabetes, stopping or modifying immunosuppressive or corticosteroid therapy is vital in the treatment of the underlying systemic disorder [11]. There are dangerous infections that dental surgeons treating COVID-19 patients should be aware of. The diagnosis of Mucor mycosis necessitates a high level of clinical suspicion. Mucor mycosis and post-covid oral maxillofacial problems can be improved with early diagnosis and prompt treatment. Mucor mycosis can have a variety of symptoms depending on the pathogen. Oral Mucor mycosis should be suspected in individuals with weakened immune systems, uncontrolled diabetes or post-covid instances, or those using immunosuppressive medicines, if the above-mentioned clinical manifestations are observed [12].

In light of its ability to mimic a wide range of conditions, from a benign infection to a metastatic tumor, actinomycosis has been dubbed the “chameleon of head and neck pathology [13].

Actinomycosis is seldom linked with regional lymphadenopathy, Due to the size of the actinomycoses organism, they cannot be transported through the lymphatic channels. This is an important feature distinguishing actinomycosis from malignancy. The CT and MR features are not very specific for the sole diagnosis of actinomycosis infection; however, characteristic features of chronic osteomyelitis can be recognized on imaging, which in the absence of aggressive bony destruction and soft tissue mass along with no cervical lymphadenopathy can fairly rule out any sinister lesion [14].

Surgery and adequate antibiotic treatment are necessary to treat actinomycosis. There must be a lengthy course of therapy, with regular checks on antibiotic sensitivity and response. Infection may return after a period of inactivity; thus, long-term follow-up is necessary [[1], [2], [3]]. In our case after analyzing the spectrum of both diseases there was decreased lymphocyte count following COVID-19 infection as well as heavy immunosuppression administration which led to invasive actinomycosis infection involving the rare site - maxilla. The additional presence of necrotic tissue further aided in actinomycotic spread locally. The present case points towards an interesting relationship between a rare infection and its association with COVID-19 and its treatment [15].

## 3 Conclusion

We conclude a positive causal association between COVID-19 and actinomycosis. Maxillary osteomyelitis, a very rare infection, and in our case, the causative organism was *Actinomyces*, which makes it even more unlikely. Patients who have been infected with COVID-19 should be tested for actinomycotic infection, which may be the only or a contributing factor to their recurring or chronic oral infections. For the sake of this patient's health, it's important to bear in mind that actinomycosis may masquerade as a head and neck illness.

## Provenance and peer review

Not commissioned, externally peer reviewed.

## Please state any conflicts of interest

Nill.

## Please state any sources of funding for your research

Nill.

## Ethical approval

Ethical approval was obtained from the ethical committee of KRL Hospital Islamabad, Ref ERC: KRL–HI–ERC/June 05/19-5.

## Consent

Obtained from the patient.

## Author’s contribution

1. The main concept was determined by Wajiha Arshad.

2. Collection of data is done by Mahjabeen Mahmood Kamal.

3. Writing of the manuscript is done by Hassan Mumtaz, Zunaira Rafique.

4. Manuscript editing is done by Manahil Rahat.

## Registration of research studies

1. Name of the registry:

2. Unique Identifying number or registration ID:

3. Hyperlink to your specific registration (must be publicly accessible and will be checked):

## Guarantor

Wajiha Arshad & Zunaira Rafique.
